# No Evidence of Superior Graft Remodeling and Maturation When Adding a Lateral Extra-articular Procedure to Anterior Cruciate Ligament Reconstruction: A Systematic Review and Meta-analysis of Comparative Studies

**DOI:** 10.1177/03635465251376662

**Published:** 2026-01-04

**Authors:** Riccardo D’Ambrosi, Edna Skopljak, Domenico Albano, Carmelo Messina, Salvatore Gitto, Francesca Serpi, Luca Maria Sconfienza

**Affiliations:** † IRCCS Ospedale Galeazzi–Sant’Ambrogio, Milan, Italy; ‡ Department of Biomedical Sciences for Health, University of Milan, Milan, Italy; § Department of Orthopedic Surgery, University Medical Centre, Ljubljana, Slovenia; ‖ Faculty of Medicine, University of Ljubljana, Ljubljana, Slovenia; ¶ Department of Biomedical, Surgical and Dental Sciences, University of Milan, Milan, Italy; # UOC Radiodiagnostica, ASST Centro Specialistico Ortopedico Traumatologico Gaetano Pini-CTO, Milan, Italy; Investigation performed at IRCCS Ospedale Galeazzi–Sant’Ambrogio, Milan, Italy

**Keywords:** ACL, anterolateral ligament, SNQ, graft maturation, magnetic resonance imaging

## Abstract

**Background:**

The use of postoperative magnetic resonance imaging (MRI) to investigate the effect of concomitant lateral extra-articular procedures (LEAPs) is not well documented in the literature. Few studies have assessed the MRI signal intensity—measured by the signal-to-noise quotient (SNQ)—of the anterior cruciate ligament (ACL) graft between individuals who underwent ACL reconstruction (ACLR) with simultaneous LEAP and those who did not. These comparative studies have produced conflicting results, though, which makes the topic particularly relevant.

**Purpose:**

To systematically compare existing evidence on graft maturation by performing a meta-analysis of combined ACLR and LEAP versus isolated ACLR.

**Study Design:**

Systematic review and meta-analysis; Level of evidence, 3.

**Methods:**

The methodology adhered to the PRISMA (Preferred Reporting Items for Systematic Reviews and Meta-Analyses) guidelines. The PubMed, Embase, and Cochrane Library databases were searched to identify potentially relevant comparative studies that analyzed postoperative graft maturation, at least 10 months after surgery, using MRI with SNQ after isolated ACL or ACL plus LEAP. The Methodological Index for Non-Randomized Studies was used for quality assessment.

**Results:**

A total of 542 patients were included, with 307 receiving isolated ACLR and 235 undergoing combined ACL plus LEAP. Meta-analysis revealed no statistical difference between the groups regarding SNQ (*P* = .574), with a mean difference of −0.58 (95% CI, –2.62 to 1.45). Neither the rank correlation test nor the linear regression test indicated any funnel plot asymmetry (*P* = .272 and *P* = .642 respectively).

**Conclusion:**

Adding a lateral extra articular procedure to ACLR does not improve graft remodeling and maturation. Furthermore, graft maturation is not influenced by the time from surgery, age, or sex. Despite the limited number of studies considered, these findings suggest that a lateral extra-articular procedure does not play a significant role in graft maturation and should be performed selectively.

After an anterior cruciate ligament (ACL) injury, the gold standard for restoring knee joint stability, minimizing long-term degenerative effects, and enhancing overall knee function is ACL reconstruction (ACLR). Although isolated ACLR has a significant success rate, 14% to 25% of cases exhibit residual rotational instability associated with unsatisfactory outcomes and graft failures.^12,14,20,36^ Therefore, effective results of ACL surgery depend on achieving rotational control of the knee.^6,13,21^

Given ACL insufficiency, the anterolateral ligament (ALL) of the knee has recently garnered attention for its potential role in enhancing the restoration of native knee biomechanics.^26^ Anatomic and biomechanical studies have shown that the ALL is crucial for assisting the ACL in managing anterolateral rotational laxity. To address residual rotational instability, extra-articular procedures such as ALL reconstruction (ALLR) or lateral extra-articular tenodesis (LET) combined with ACLR have been increasingly utilized.^26^

In a biomechanical in vitro setting, a comparison of 5 anterolateral procedures revealed that the addition of either ALLR or the modified Ellison procedure restored overall native knee kinematics in a combined ACL plus anterolateral–deficient knee. Superficial and deep Lemaire and modified MacIntosh tenodeses achieved excellent rotational control but overconstrained internal rotation, leading to a change from intact knee kinematics.^28^

Many studies suggest that combining ACL and lateral extra-articular procedure (LEAP) techniques improves rotational stability and patient-reported outcomes compared with isolated ACLR. Reliable restoration of normal knee kinematics and load-sharing with the ACL graft helps explain these benefits.^32^ Despite the abundance of clinical results of contemporary ACLR, the use of postoperative magnetic resonance imaging (MRI) to investigate the effect of concomitant LEAP is not well documented in the literature.

The signal-to-noise quotient (SNQ) is widely used to explore the effect of risk variables on graft ligamentization in ACLR; however, there are little data on the time to ligamentization based on the graft’s SNQ. Radiological ligamentization was thought to be complete 12 months after ACLR.^39^ However, recent longitudinal MRI studies have implied that ligamentization takes longer. After ACLR, SNQ increased at 4 to 6 months, gradually declined, and plateaued at 18 to 24 months.^39^ Furthermore, the extent of SNQ indicative of the completion of ligamentization remains unidentified.^39^

Few studies have evaluated the MRI signal intensity (SI)—measured by SNQ—of the ACL graft between individuals who received ACLR with simultaneous LEAP and those who did not. These comparative studies have produced contradicting results, making the topic particularly relevant, and it is still debated whether the improved outcomes are the result of the influence of concomitant ALLR on ACL graft healing.^2,19,33,38,40,41^

The aim of this study was to perform a systematic review and meta-analysis of the existing evidence on postoperative ACL graft maturation, evaluated via MRI with SNQ, comparing combined ACLR and LEAP with isolated ACL.

## Methods

A systematic search strategy was developed according to PRISMA (Preferred Reporting Items for Systematic Reviews and Meta-Analyses) guidelines and is registered in the PROSPERO registry (CRD420250652697).^27,30^

An electronic database search was conducted to identify potentially relevant research articles that analyzed graft maturation after ACLR with or without an associated anterolateral procedure. The MEDLINE (PubMed), Embase (Elsevier), and Cochrane Library databases were searched on February 15, 2025, and 2 weeks later, using the following Boolean search terms: “ACL reconstruction” OR “anterior cruciate ligament reconstruction” OR “ACL” AND “anterolateral” OR “extra-articular” OR “ALL” OR “LEAP” OR “ALLR” OR “Lemaire” OR “Ellison” OR “Cocker-Arnold” AND “graft maturation” OR “MRI” OR “remodeling” OR “magnetic resonance imaging” OR “SNQ” OR “signal-to-noise-quotient” OR “signal-to-noise-ratio” OR “SNR.”

### Eligibility Criteria

The literature selected for this study was based on the below criteria.

#### Study Design

Only comparative studies were included in the systematic review and meta-analysis.

#### Participants and Interventions

Comparative studies were conducted in skeletally mature patients who underwent isolated ACLR or ACLR plus LEAP and were evaluated for postoperative graft maturation, at least 10 months after surgery, using MRI with SNQ.^39^

#### Type of Outcome Measures

The primary extracted and recorded outcome measure was the SNQ of the new graft. Lower SNQ ratios indicate less water content and theoretically better maturity and healing of the graft.

### Data Collection and Analysis

#### Study Selection

The retrieved articles were first screened by title and, if found relevant, further screened by reading the abstract. After excluding studies that did not meet the eligibility criteria, the entire content of the remaining articles was assessed for eligibility. To minimize the risk of bias, we reviewed and discussed all selected articles, references, and articles excluded from the study. In case of disagreement among the reviewers, the senior investigator (L.M.S.) made the final decision. At the end of the process, additional studies that might have been missed were searched manually by reviewing the reference lists of the included studies and relevant systematic reviews.

#### Data Collection Process

Data were extracted from the selected articles by the first 2 authors (R.D. and E.S.) using a computerized tool created with Microsoft Access (Version 2010; Microsoft Corp). Each article was validated again by the first author (R.D.) before analysis. For each study, data on patient return to sports and rerupture rate were extracted.

#### Level of Evidence

The levels of evidence set by the Oxford Centre for Evidence-Based Medicine were used to categorize the level of evidence.^24^

#### Evaluation of the Quality of Studies

The quality of the selected studies was evaluated using the Methodological Index for Non-Randomized Studies (MINORS) score. The checklist includes 12 items, of which the last 4 are specific to comparative studies. Each item was assigned a score of 0 to 2 points. The ideal score is 16 points for noncomparative studies and 24 points for comparative studies.^34^

### Statistical Analysis

A meta-analysis was conducted on the mean difference of SNQ, calculated as the difference between the mean SNQ in ACL plus LEAP and the mean SNQ in isolated ACL (ie, ACL used as the reference group). A random-effects model was estimated using the restricted maximum-likelihood estimator for the variance. Between-study variations were assessed with the Cochran Q chi-square test of heterogeneity and the *I*^2^ statistic. Statistical heterogeneity was defined as substantial if *I*^2^ >50%.^15^

Subgroup analyses were conducted to explore the mean difference of SNQ by age, time from surgery, and male-to-female ratio. Age and time from surgery were included as binary variables, specifically mean age <30 years versus ≥30 years and mean time from surgery <500 days versus ≥500 days. Both mean age and time from surgery were weighted for the number of patients in each group (ACL and ACL plus LEAP).

The male-to-female ratio was included as a binary variable (number of women equal to or greater than the number of men: yes/no); however, a study was excluded from this analysis because data on sex were not available from the primary study. Potential publication bias and small-study effects were assessed through the funnel plot, as well as through the rank correlation test and the regression test on funnel plot asymmetry. All tests were 2-tailed. A *P* value <.05 was considered to indicate statistical significance. The analysis was carried out using R (Version 4.3.0; R Foundation for Statistical Computing; https://www.R-project.org/), specifically with the meta (Version 8.0.1) and metafor (Version 4.2.0) packages.

## Results

**Figure 1.**
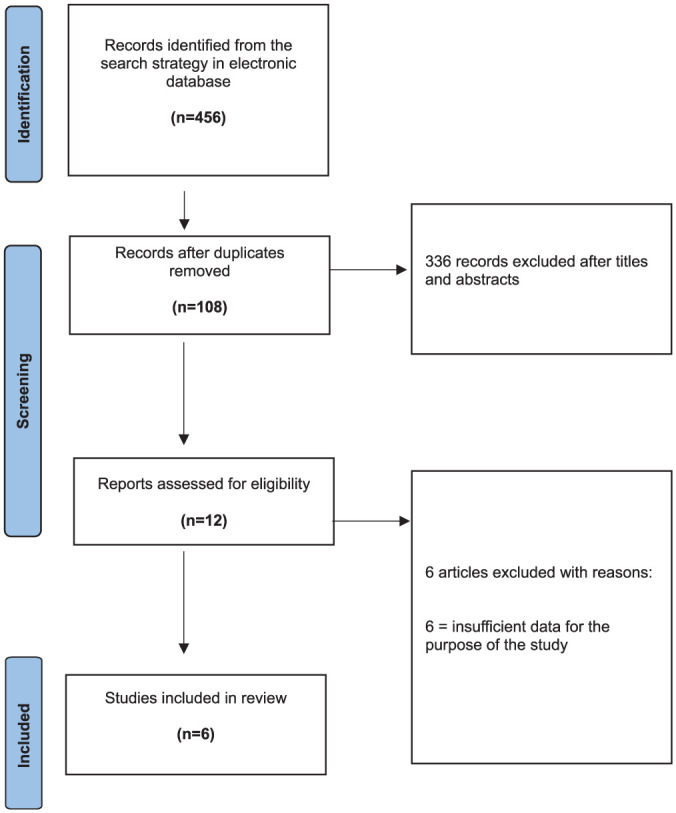
PRISMA (Preferred Reporting Items for Systematic Reviews and Meta-Analyses) flowchart indicating research article inclusion for final analysis.

The initial search of the 3 electronic databases yielded 456 records. The titles and abstracts of 108 studies were reviewed after eliminating 336 duplicates. After title, abstract and full-text review, 12 articles were assessed for eligibility. Finally, the reviewers excluded 6 records after evaluating the full texts, and 6 articles were included in the final analysis of this review.^2,19,33,38,40,41^ The PRISMA diagram is shown in Figure 1.

### Study Characteristics

A total of 6 comparative studies met the inclusion criteria and were included in the final analysis. All studies had a level of evidence of 3. The mean MINORS score was considered good (16 ± 0.6 points). A total of 542 patients were included, with 307 receiving isolated ACLR and 235 undergoing combined ACL plus LEAP. Details of the included studies are reported in Table 1.

**Table 1:** Characteristics of Patients and Studies*^a^*

Study	MINORS Score	No. of Patients	Sex, Male/ Female	Age, yrs	Graft	Surgical Technique	LEAP	Time From Surgery to MRI, days	SNQ
Isolated ACL									
Cavaignac et al, 2020^2^	15	31		33.1 ± 8.3	Quadrupled ST	Inside-out		405.0 ± 60.7	5.9 ± 3.7
Rojas et al, 2021^32^	16	30	13/17	29.7 ± 8.2	Hamstrings	Anteromedial portal		300	4.62 ± 4.29
Yang et al, 2025^36^	17	122	100/22	28.7 ± 12.0	Quadrupled hamstrings	Inside-out		1023 ± 222	4.7 ± 3.5
Yau and Lin, 2024^38^	16	40	40/0	27 ± 6.3	Quadrupled hamstrings	Anteromedial portal		501 ± 102	5.2 ± 4.8
Ye et al, 2022^39^	16	44	31/13	29.3 ± 8.6	Quadrupled hamstrings	Transtibial technique		730	9.35 ± 5.01
Jacob et al, 2024^18^	16	40	23/17	18.4 ± 3.6	Quadrupled ST	Anteromedial portal and inside-out reaming technique		405 ± 182	9.5 ± 11.4
ACL + LEAP									
Cavaignac et al, 2020^2^	15	31		27.2 ± 6.7	Quadrupled ST	Inside-out	LET with gracilis tendon folded into 2	349.0 ± 39.0	2.6 ± 4.9
Rojas et al, 2021^32^	16	22	10/12	31.1 ± 6.1	ST/GR	Anteromedial portal	Modified Lemaire with central part of the fascia lata	300	7.59 ± 4.68
Yang et al, 2025^36^	17	54	44/10	26.7 ± 10.6	Quadrupled hamstring	Inside-out	ALL reconstruction with fresh-frozen tibialis anterior allograft	1092 ± 270	2.8 ± 1.6
Yau and Lin, 2024^38^	16	40	40/0	26.2 ± 6.0	Quadrupled hamstring	Anteromedial portal	ALL reconstruction with ITB or ST	480 ± 102	6.9 ± 4.6
Ye et al, 2022^39^	16	48	36/12	29.3 ± 7.7	Quadrupled hamstring	Transtibial technique	Anterolateral augmentation with gracilis + anterior half of the peroneus longus tendon	730	7.43 ± 4.39
Jacob et al, 2024^18^	16	40	23/17	18.7 ± 3.8	Quadrupled ST	Anteromedial portal and inside-out reaming technique	Modified deep Lemaire with ITB	390 ± 98	9.0 ± 14.9

a ACL, anterior cruciate ligament; ALL, anterolateral ligament; GR, gracilis; ITB, iliotibial band; LEAP, lateral extra-articular procedures; LET, lateral extra-articular tenodesis; MINORS, Methodological Index for Non-Randomized Studies; MRI, magnetic resonance imaging; SNQ, signal-to-noise-quotient; ST, semitendinosus.

### Signal-to-Noise Quotient

**Figure 2.**
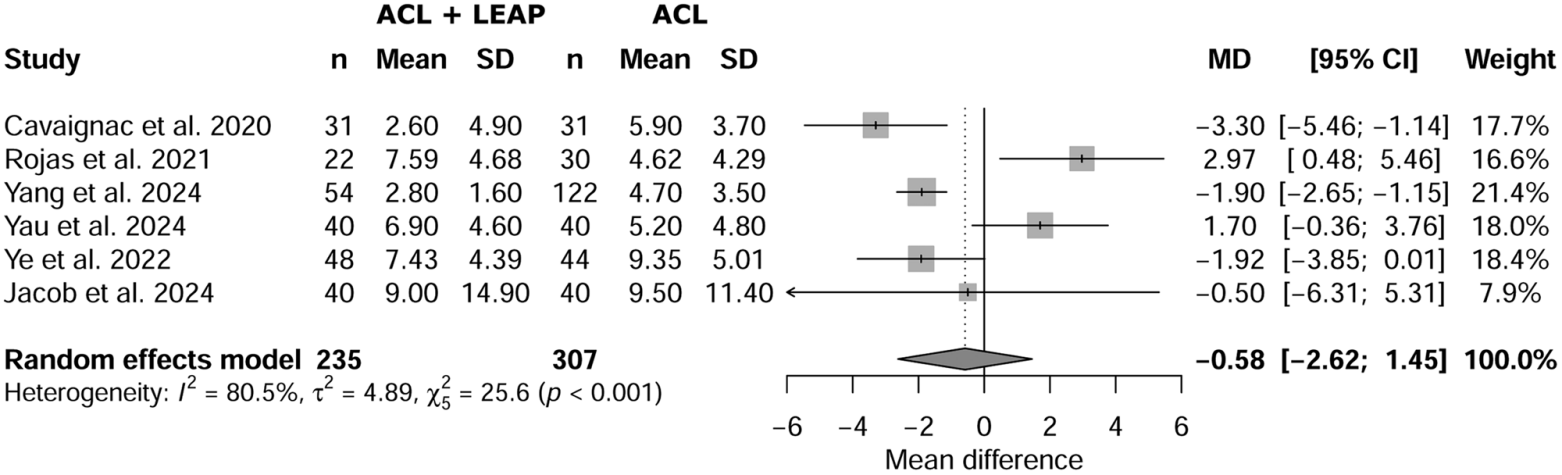
Forest plot of signal-to-noise-quotient between isolated anterior cruciate ligament (ACL) and ACL reconstruction plus lateral extra-articular procedure (LEAP).

Meta-analysis showed no statistical difference between the groups regarding SNQ (*P* = .574), with a mean difference of −0.58 (95% CI, –2.62 to 1.45). The forest plot in Figure 2 illustrates SNQ between the 2 groups.

### Funnel Plot

**Figure 3.**
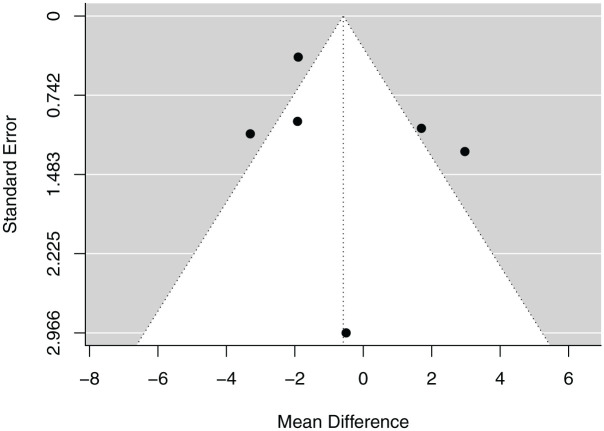
Funnel plot demonstrating the symmetry of the included studies regarding rank correlation and linear regression on signal-to-noise-quotient.

Neither the rank correlation test nor the linear regression test indicated any funnel plot asymmetry (*P* = .272 and*P* = .642, respectively). A funnel plot of the estimates is shown in Figure 3.

### Age

**Figure 4.**
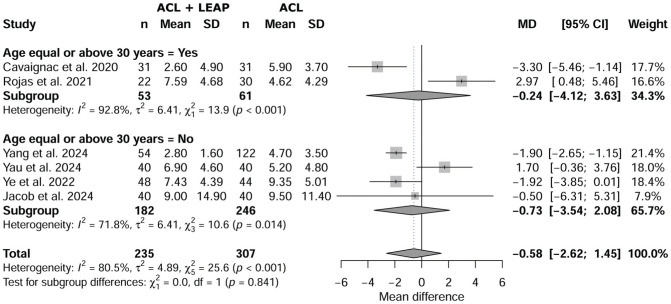
Effect of age on signal-to-noise-quotient between isolated anterior cruciate ligament (ACL) and ACL reconstruction plus lateral extra-articular procedure (LEAP).

Meta-regression revealed no significant effect of age on the SNQ (*P* = .841) between ACL plus LEAP and isolated ACL, with a mean difference of −0.58 (95% CI, –2.62 to 1.45) (Figure 4).

### Time From Surgery

**Figure 5.**
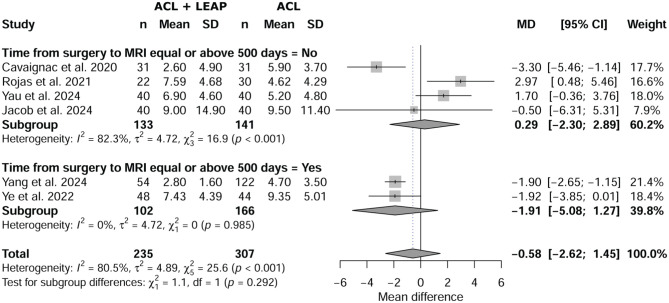
Effect of time from surgery on signal-to-noise-quotient between isolated anterior cruciate ligament (ACL) and ACL reconstruction plus lateral extra-articular procedure (LEAP).

Meta-regression revealed no significant effect of time from surgery on SNQ (*P* = .292) between ACL plus LEAP and isolated ACL, with a mean difference of −0.58 (95% CI, –2.62 to 1.45) (Figure 5).

### Sex

**Figure 6.**
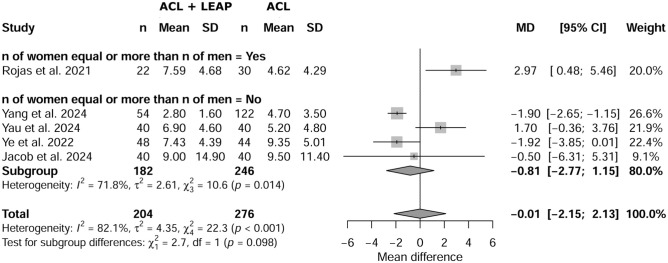
Effect of sex on signal-to-noise-quotient between isolated anterior cruciate ligament (ACL) and ACL reconstruction plus lateral extra-articular procedure (LEAP).

Meta-regression showed no significant effect of sex on SNQ (*P* = .098) between ACL plus LEAP and isolated ACL, with a mean difference of −0.01 (95% CI, –2.15 to 2.13) (Figure 6).

## Discussion

The most significant findings of the current systematic review and meta-analysis reveal that there is no difference in graft maturation and remodeling when adding an anterolateral procedure to ACLR; furthermore, graft maturation is not affected by the time from surgery, age, or sex.

The purpose of this article was to shed light on an extremely topical and interesting theme, in which, as the included articles show, there has never been unanimity of opinion. Graft maturation is a complex process with many unknown steps that could help improve ACLR.^5^

First, an important finding is that a free tendon graft, when implanted in the human knee to replace a ruptured ACL, survives in the intra-articular environment. At any given time point after reconstruction, the ACL is histologically viable, with evidence of nourishing vascularization at least in the graft’s periphery and no signs of significant necrosis. The origin of the neovascularization is thought to be the Hoffa fat pad and the synovium, although no report provides definitive evidence to support this hypothesis.^5^

The research consistently outlines a process whereby implanted grafts gradually lose their “tendon-specific” biological characteristics while increasingly displaying “ligamentous” histological traits. This ligamentization process represents a continuum of biological changes rather than a sequence of discrete, temporally dependent biological events. Dividing this process into distinct stages is deemed beneficial, particularly concerning postoperative rehabilitation methods and the timing of return to preinjury athletic activity. The existing literature lacks consensus on the time frames for ligamentization, revealing significant discrepancies among various authors regarding the specified time points.^5^

The studies included in the current systematic review and meta-analysis used MRI to assess and evaluate graft maturation over time. In the 1990s, Howell et al^17^ were among the first to propose an MRI grading system that categorized the signal measured in the ACL graft as low, intermediate, or high, where lower SI was assumed to indicate a more mature reconstructed graft. Nonetheless, SI in typical weighted MRI studies is expressed in relative units that depend on acquisition parameters and scanner attributes. Consequently, SI does not provide absolute quantification of biophysical tissue characteristics. To standardize the graft SI grayscale value, numerous studies have used the SNQ to evaluate graft maturity by MRI, a technique initially introduced by Stöckle et al.^31,35^

Regrettably, conventional techniques typically used to assess SI with single-coil imaging configurations are rendered invalid when utilizing multichannel coils and parallel imaging methodologies. The orientational fluctuation in MRI signals, known as the magic angle effect (MAE), must also be considered when assessing the ACL graft. The range of circumstances in which a substantial MAE can occur is often undervalued. Furthermore, SIs are hardly comparable among different scanners, making generalization of this parameter impossible.30

In 2019, Van Dyck et al^36^ conducted a systematic review of studies that have used MRI SI as a proxy for ACL graft maturity and to identify potential confounding factors in assessing the ACL graft in conventional MRI studies. The authors concluded that the MRI acquisition and evaluation methods used to assess ACL grafts are highly heterogeneous, impeding comparisons of SI between successive scans and across independent studies.^36^

A similar study was performed by Panos et al,^31^ who aimed to quantitatively analyze reports of serial MRI of the ACL graft during the first year after ACLR. MRI signal varies with graft type, graft source, and time after ACLR. Enhanced graft maturity during the first postoperative year was associated with hamstring autografts, with and without remnant preservation. Serial MRI during the first postoperative year may be clinically useful for identifying biologically or mechanically deficient ACL grafts at risk for failure.^31^ Hence, assessing ACL graft maturity before returning to sports is of high clinical interest, and MRI has been shown to be a feasible option. However, current studies evaluating the MRI signal changes of ACL autografts and their correlation to clinical outcomes and knee laxity remain inconclusive.^8,10^

Lutz et al^25^ investigated the ACL autograft maturation process via MRI over 2 years postoperatively, comparing it to a native ACL signal and correlating the results with clinical outcomes, return to preinjury sports levels, and knee laxity measurements. The ACL graft signals became hyperintense 6 months postoperatively and approximated the signal of a native intact ACL at 12 and 24 months.^25^ Patients with a hypointense ACL graft signal at 2 years of follow-up were more likely to return to preinjury sports levels.^25^

In our systematic review, there was no evidence that factors such as age and sex can affect graft maturation. Recent literature has analyzed several factors that may influence graft maturation, including surgical technique, graft type, and graft angle. Hooghof et al^16^ found that no differences in SI of the graft on MRI were seen between the femoral drilling techniques 1 year after ACLR, suggesting similar graft maturation at that time. In contrast, Ahn et al^1^ revealed that patients who underwent anatomic femoral tunnel placement showed a higher graft maturation score on second-look arthroscopy, along with better clinical outcomes, than patients who underwent nonanatomic femoral tunnel placement. Regarding the graft, Fukuda et al^11^ demonstrated that patellar tendon maturation is superior to that of double-bundle hamstring based on morphological and MRI evaluations after anatomic ACLR, although no significant differences were found in clinical scores.

What seems to positively affect graft maturation is the graft bending angle (GBA), the angle between the femoral bone tunnel and the line connecting the femoral and tibial tunnel apertures. The GBA has been shown to influence stress within the graft, and it might be an important factor in graft healing within the joint and bone tunnel. A higher GBA could lead to tunnel widening and has been found to correlate significantly with a higher graft SI on MRI, which may indicate poorer ACL graft healing.^3,22,23^

All the comparative studies included in the meta-analysis adopted the SNQ to evaluate graft maturation. Reports of the SNQ used in the analysis of ACLR of the knee in the literature show good interrater reliability, ranging from 0.72 to 0.89 depending on the study, as well as good intrarater reproducibility, ranging from 0.71 to 0.88.^9^

In recent years, other procedures have been performed to evaluate graft maturation with good to excellent results. In fact, in 2019, Chu and Williams^4^ evaluated quantitative MRI UTE-T2* and T2 to assess graft maturation. UTE-T2* mapping suggested substantial changes within the graft during the first 6 months after surgery. T2* and UTE-T2* mapping showed relatively stable graft composition from 6 months to 1 year, consistent with remodeling, followed by decreases from 1 to 2 years, suggestive of continuing maturation. MRI UTE-T2* and T2* mapping demonstrated potential clinical utility as noninvasive quantitative imaging metrics for evaluating human ACL grafts.^4^

Niki et al^29^ examined whether the T1rho and T2 maps reflect graft function and maturation after ACLR. The T1rho sequence was able to visualize the tendinous portions of the anteromedial bundle and posterolateral bundle more clearly than the T2 map sequence, even on grayscale images. Mean T1rho and T2 map values gradually decreased during the first operative year, but the trend was more prominent and consistent for T1rho values than for T2 map values.^29^ Correlation analysis revealed that T1rho and T2 map values at 1 year correlated significantly with anteroposterior laxity at 2 and 4 years.^29^

The rationale for adding anterolateral procedures to ACLR has been analyzed in several biomechanical studies. In fact, adding LEAP procedures has been shown to restore knee kinematics. These procedures decrease the strain on the ACL graft while increasing rotatory knee stability.^29^ A recent study demonstrated that augmentation of ACLR by LET decreased the ACL graft force by 80%. A similar decrease in load on the graft was observed at 30° of knee flexion in a simulated setup.^26^

At the clinical level, a question that often arises is whether adding extra surgical time may entail risks associated with the procedure. In this scenario, D’Ambrosi et al^7^ recently demonstrated that adding a LEAP, regardless of the surgical technique, can reduce the failure rate without increasing the number of complications at midterm follow-up.

### Limitations

Several strengths and limitations of this systematic review and meta-analysis must be considered. This study’s strength lies in being the first to analyze exclusively comparative studies about graft maturation comparing isolated ACLRs and ACLR combined with LEAP.

Previous systematic reviews and meta-analyses in the literature included older studies with lower levels of evidence and higher risk of bias. The limitations of the study are related to the limited number of studies with different study designs evaluated. The heterogeneity of studies, including ACLR graft selection, LEAP graft selection, attachment points, and surgical techniques, limits direct comparisons when evaluating clinical outcomes. Furthermore, concomitant surgery was not considered an exclusion criterion. Therefore, it is important that future studies specify the length of follow-up, the graft used, and any concomitant surgeries performed.

## Conclusion

Adding a lateral extra-articular procedure to ACLR does not improve graft remodeling and maturation. Furthermore, graft maturation is not affected by the time from surgery, age, or sex. Despite the limited number of studies considered, our findings suggest that a lateral extra-articular procedure procedure does not play a significant role in graft maturation and should be performed selectively.
